# Diverse Sources of Social Support and Cognitive Functioning by Race, Ethnicity, and Nativity

**DOI:** 10.1093/geroni/igaa057.935

**Published:** 2020-12-16

**Authors:** G Robin Gauthier, Marc Garcia, Catherine García

**Affiliations:** 1 University of Nebraska-Lincoln, Lincoln, Nebraska, United States; 2 University of Nebraska, Lincoln, Nebraska, United States; 3 University of Southern California, Los Angeles, California, United States

## Abstract

This study examines the relationship between social support profiles and cognitive functioning by race, ethnicity and nativity in older adults using cross-sectional data drawn from the Health and Retirement Study (2010 and 2012). We employed a hierarchical clustering routine to generate nine support profiles that differentiated three sources of support: children, wider family relationships and friendships. Cognitive functioning was measured as the score on the Telephone Interview for Cognitive Status (TICS-m), a 27 point scale of cognitive function. Our approach explicitly acknowledges the ambivalence and multidimensionality of close relationships and the resources embedded within them. Descriptive analyses revealed significant differences in access to support across demographic groups. White respondents are over-represented in profiles that are characterized by support from friends, and under-represented in family support profiles. The reverse is found among Foreign-born Hispanic respondents who are over-represented in the profiles characterized by high family support and under-represented in those with high friend support. Native-born Hispanic respondents and Black respondents have less clear support patterns, although both are more likely to receive support from family and children compared to friends. Findings from the poisson regression suggest that the relationship between familial support and cognitive decline is stronger among Hispanic respondents, particularly those who are foreign born. These findings are supported even with the inclusion of other relationship quality indicators including negative support and frequency of contact.

